# *Streptomyces* Differentiation in Liquid Cultures as a Trigger of Secondary Metabolism

**DOI:** 10.3390/antibiotics7020041

**Published:** 2018-05-14

**Authors:** Ángel Manteca, Paula Yagüe

**Affiliations:** Área de Microbiología, Departamento de Biología Funcional IUOPA, Facultad de Medicina, Universidad de Oviedo, 33006 Oviedo, Spain; mantecaangel@uniovi.es

**Keywords:** *streptomyces*, screening, antibiotics, secondary metabolism, differentiation, elicitors, morphology, liquid cultures

## Abstract

*Streptomyces* is a diverse group of gram-positive microorganisms characterised by a complex developmental cycle. *Streptomycetes* produce a number of antibiotics and other bioactive compounds used in the clinic. Most screening campaigns looking for new bioactive molecules from actinomycetes have been performed empirically, e.g., without considering whether the bacteria are growing under the best developmental conditions for secondary metabolite production. These screening campaigns were extremely productive and discovered a number of new bioactive compounds during the so-called “golden age of antibiotics” (until the 1980s). However, at present, there is a worrying bottleneck in drug discovery, and new experimental approaches are needed to improve the screening of natural actinomycetes. *Streptomycetes* are still the most important natural source of antibiotics and other bioactive compounds. They harbour many cryptic secondary metabolite pathways not expressed under classical laboratory cultures. Here, we review the new strategies that are being explored to overcome current challenges in drug discovery. In particular, we focus on those aimed at improving the differentiation of the antibiotic-producing mycelium stage in the laboratory.

## 1. Introduction

The *Streptomyces* genus includes an important group of biotechnological bacteria. They produce two-thirds of the antibiotics of medical and agricultural interest, several antitumor agents, antifungals, and a great number of eukaryotic cell differentiation effectors, such as apoptosis inducers and inhibitors [1]. Drug discovery from *streptomycetes* fell considerably after initial screenings where the most common compounds were discovered. Antibiotic resistance is increasing dramatically, and new antibiotics are urgently required in the clinic. Alternative methods, such as the exploration of chemical libraries and combinatorial chemistry, have provided limited yields. Screening from nature has resumed through methods such as exploring new environments, looking for elicitors, accessing the metagenome, etc.

**Figure 1:**
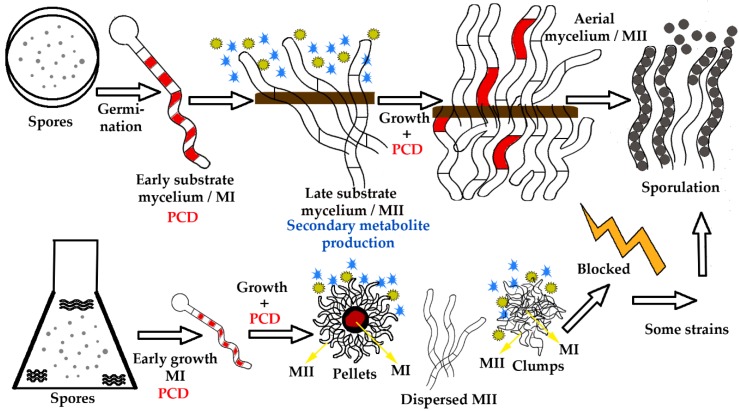
*Streptomyces* growth in solid cultures (**upper panels**) and liquid cultures (**lower panels**). In solid cultures (petri plates), spores germinate developing a compartmentalised mycelium (early substrate mycelium, MI) with 1 µm average cross-membrane spacing [6]. Some of the MI cells suffer a first round of programmed cell death PCD (red segments). The remaining viable segments start to grow as a multinucleated mycelium with sporadic septa (early MII, late substrate mycelium) [6]. The mycelium substrate suffers a second round of PCD (red segments) and differentiates into a mycelium that starts to grow into the air (the medium/agar border is indicated by a brown line) (late MII, aerial mycelium). Part of the aerial hyphae form spore chains (black circles). In liquid cultures, there is germination, MI development, PCD (in the centre of the mycelial pellets) and MII differentiation (in the periphery of the pellets). In most species, there is no aerial mycelium formation or sporulation, and hyphae form pellets and clumps [2]. Secondary metabolites (outlined as yellow circles and blue starts) are produced by the MII hyphae.

One of the most important characteristics of *Streptomyces* is its complex life cycle, which is closely related to secondary metabolite production [2] (outlined in Figure 1). In solid sporulating cultures, development starts with spore germination and the rapid development of compartmentalised hyphae into the medium (early substrate mycelium or MI) [3]. After that, programmed cell death (PCD) occurs (red cellular segments in Figure 1) which triggers the differentiation of the multinucleated (MII) antibiotic-producing hyphae (late substrate mycelium, early MII) [3,4]. Then, the mycelium starts to grow into the air forming the aerial mycelium (late MII). At the end of the cycle, there is a second round of PCD, and most of the remaining viable hyphae undergo a process of compartmentalisation that culminates in the formation of unigenomic spores [5].

Most *streptomycetes* do not sporulate in liquid cultures. Therefore, it was previously assumed that under these conditions, there was no differentiation. However, industrial antibiotic production is mostly performed in liquid cultures (flasks and bioreactors). Currently, it is known that in liquid cultures, differentiation is comparable to that observed in solid cultures (Figure 1). In liquid cultures, there is a first mycelium stage (MI), PCD and the differentiation of a secondary metabolite, producing mycelium (MII). However, in most *Streptomyces* strains, aerial mycelium formation and sporulation are blocked [6] (Figure 1). *S. coelicolor* proteomic and transcriptomic studies have shown that physiological differentiation in liquid and solid cultures is comparable [6,7]. MII expresses/translates the genes/proteins involved in secondary metabolism in both solid and liquid cultures [6,7].

Surprisingly, *Streptomyces* differentiation as a trigger for antibiotic production remains almost unexplored. The absence of a developmental model to describe differentiation in liquid cultures has inhibited the understanding of the relationship between macroscopic morphology (pellet and clump formation) and differentiation. Pellet and clump formation has been classically correlated with secondary metabolite production, but the relationship between both processes remains obscure. Most authors have affirmed that pellets and clumps are fundamental for secondary metabolite production (e.g., retamycin in *S. olindensis* [8], nikkomycins in *S. tendae* [9], hybrid antibiotics in *S. lividans* [10]), while some authors have affirmed that pellet and clump formation reduces antibiotic production (e.g., nystatin in *S. noursei* [11], tylosin in *S. fradiae* [12]). More recently, our group demonstrated that one of the key events in the activation of secondary metabolite production in *Streptomyces* liquid cultures is the differentiation of MII (e.g., actinorhodin/undecylprodigiosin production in *S. coelicolor* [2,13], microbial transglutaminase production in *S. mobarensis* [14], apigenin and luteolin production in *S. albus* [15]). The differentiation of this mycelium is conditioned by PCD of the vegetative hyphae (MI) [2], which, in liquid cultures, depends on the growth rate of the strain and hypha aggregation (pellet/clump formation) [2,7,14,15,16]. However, secondary metabolism has additional regulations (elicitors activate specific biosynthetic pathways) [17], and most *Streptomyces* strains do not display all their potential secondary metabolites under standard developmental laboratory conditions, even if they are differentiated at the MII stage [7].

Each *Streptomyces* strain can harbour up to 30 secondary metabolite pathways, but only a few of these are active in usual screening processes [18]. Activating these pathways in the lab will be crucial in the process of screening for new secondary metabolites from actinomycetes. Here, we review the most important strategies that are being explored to activate cryptic pathways and/or those that are being explored to enhance secondary metabolites production.

## 2. Screening for New Secondary Metabolites from *Streptomycetes*

The search for new actinomycetes in unexplored niches or from the screening of strains that have not been previously cultivated is useful, but usually leads to the rediscovery of already known compounds [19]. New screening strategies are necessary to overcome the current challenges of discovering new bioactive compounds [19]. In 2013, Arryn Craney et al. [20] summarised the new strategies that are being used to enhance secondary metabolite production and activate cryptic pathways, dividing them into unselective and selective methods [20]. Unselective methods are non-specific methods that are used to screen for new activities, whereas selective methods are biosynthetic cluster-specific methods that are used to improve the production of already known molecules [20].

Non-specific methods were largely used during “the golden age of antibiotics”, and they are still useful. These methods include classical strategies, such as changing media components, increasing general precursors (metabolic engineering), inducing stress responses (with heat/ethanol/salt/acid shock, nutrient limitations) [21], and obtaining strains that overproduce secondary metabolites by random mutagenesis [22,23,24]. More novel non-specific methods include ribosomal engineering (the alteration of ribosomal proteins to activate cryptic secondary metabolites in *streptomycetes*) [20,25] and the use of small molecules as elicitors of secondary metabolism [20,26] (Table 1). Differentiation of the antibiotic producer mycelium (MII) as a non-specific method to activate antibiotic production remains almost unexplored. There has been no previous analysis of the frequency of *Streptomyces* strains that do not produce secondary metabolites because they are not differentiated at the MII stage in the laboratory.

Biosynthetic cluster-specific methods include self-resistance engineering (upregulation of self-resistance genes), regulatory engineering (overexpression of activators or elimination of repressors) and genome mining to search for new biosynthetic pathways [20] (Table 2). One of the most important biosynthetic cluster-specific methods is heterologous expression. Heterologous expression has been used to express *Streptomyces* industrial enzymes, such as laccases, in microorganisms with simpler developmental cycles than *Streptomyces*, such as *E. coli* [27]. However, the complex biosynthetic pathways of *Streptomyces* rarely can be expressed in simple expression hosts, such as *E. coli* or *Bacillus*. Thus, other *streptomycetes*, such as *S. lividans*, *S. albus*, *S. coelicolor* or *S. avermitilis*, are commonly used as expression hosts [28]. The activation of cryptic metabolites through the expression of the *Streptomyces coelicolor* pleiotropic regulator, *Afs*Q, in other *streptomycetes* [29] has been successfully achieved. Combinatorial biosynthesis, chemical modification of existing molecules, has been largely developed over the last 20 years, in particular, progress has been made in the last few years thanks to genome mining and synthetic biology [30,31,32]. Differentiation of *Streptomyces* MII was successfully used to enhance the production of various products [2,13,14,15] through its role as a trigger for antibiotic production (described in Section 2.3).

### 2.1. Streptomyces Differentiation Strategies Based on Elicitors

In the last few years, effort has been made to elucidate the mechanism by which some small molecules (elicitors) affect differentiation and secondary metabolite production in *Streptomyces* strains. Elicitors can be defined as diffusible signals that are able to induce cryptic pathways and/or differentiation in *Streptomyces* cultures [17]. Some elicitors act as signals for interspecies interaction [33]. Thus, subinhibitory concentrations of certain antibiotics produced by a given *Streptomyces* strain accelerate differentiation and antibiotic production in other *Streptomyces* strains through “pseudo” gamma-butyrolactone receptors [33]. Another good strategy is the use of random chemical probes (natural or synthetic) as elicitors (reviewed in [21]).

One of the most common strategies used to activate secondary metabolism and differentiation is mimicking the ecological environment through co-cultures of different microbes [17,34]. This methodology typically uses species that have symbiotic relationships with *Streptomyces* in nature [35,36] or pathogen partners that activate the production of antimicrobial compounds [37,38,39]. For instance, fungal elicitors (complex mix of cell walls and filtered cultures) positively affect the production of natamycin [40], bacterial and yeast elicitors improve valinomycin production [41], nutrients such as glucose and xylose repress the production of actinorhodin [42,43], and small molecules, such as GlcNAc or phosphate, can trigger differentiation and antibiotic production in *S. coelicolor* through the activation of *act*II-ORF4/*red*Z genes [44].

Pimentel-Elardo et al. [45] developed an activity-independent screening method based on the use of elicitors, to prevent the rediscovery of the most active/abundant compounds. In addition, cheminformatics techniques are used to identify the putative biological activities of identified compounds [45]. The use of elicitors increases the production of low-abundant compounds which were undetected in the classical activity dependent screening. The chemical elicitor “CI-ARC” has been identified as being responsible for triggering several cryptic biosynthetic genes [45].

### 2.2. Differentiation Strategies Based on Macroscopic Morphology

#### 2.2.1. The Genetic Control of Aggregation and Macroscopic Morphology in Liquid Cultures

Large-scale antibiotic production is mostly performed in liquid cultures. It is almost unanimously accepted that the macroscopic morphology of the mycelium (pellets and clump formation) is correlated with the production of secondary metabolites. However, it was not until recently that the genes controlling pellet and clump formation have been characterised. The *S. coelicolor mat* gene cluster [46] and the *cslA*, *glxA*, *dtpA* genes [47,48,49] are responsible for mycelial aggregation and pellet formation. These genes could be a great tool for controlling the morphology in industrial fermentation.

The *Streptomyces* life cycle in liquid cultures starts with the germination of spores. Awakening from the dormant spore state depends on the level of AMPc in the cultures [50] and involves the small hydrophobic protein NepA, [51]. The expression of several sigma factors involved in osmotic and oxidative stress (SigH, SigB, SigI, SigJ) undergoes remarkable changes during germination, indicating that germination evokes stress-like cell responses [52]. Several genes encoding proteins involved in lipid metabolism and membrane transport are overexpressed during germination [52]. The conservation of d-alanyl-d-alanine carboxypeptidase (SCO4439) contributes to the swelling phase of germination [53]. Cell wall hydrolases participate in germination [54]. SsgA protein marks the germinative tube emission points [55]. Recently it was described that during germination, spores aggregate due to extracellular glycans synthesized by the MatA, MatB [46,56] and the CslA/GlxA/DtpA proteins [56]. These aggregates determine the macroscopic morphology (pellets and clumps) of the culture [56] which triggers PCD and the physiological differentiation of the antibiotic producer, mycelium MII [2].

Another issue that influence secondary metabolite production is sporulation. Several *streptomycetes* are able to sporulate in liquid cultures [57] and some strains, that normally do not sporulate are also able to sporulate in bioreactors due to the stress generated in the fermenter [13]. Sporulation stops metabolism, including secondary metabolite production. Consequently, in industrial fermentations and during screening for new secondary metabolites, it is important to avoid sporulation to increase and maintain secondary metabolism for as long as possible [13].

#### 2.2.2. Monitoring of *Streptomyces* Macroscopic Morphology and Differentiation in Liquid Cultures

Pellet and clump formation led to differentiation and secondary metabolism [2]. Consequently, new methodologies to monitor macroscopic morphology have been developed. Laser diffraction has been used to measure pellet size [58]. Flow cytometry has been used to establish pellet size distribution of culture populations [59,60]. Recently, a useful algorithm was developed as a plug-in for the open-source software, ImageJ, to characterize the morphology of filamentous microorganisms in liquid cultures [61]. Mathematical models have been performed to predict the behaviour of *Streptomyces* liquid cultures based on pellet/clump morphology [62,63].

Biophysical parameters (e.g., pH, viscosity, agitation, dissolved oxygen levels and surface tension, among others) directly affect morphology and differentiation [13,64]. These parameters must be considered when scaling up production to industrial conditions [65]. Interestingly, a recent study downscaled liquid cultures to the 100 µL scale in microtiter plates [66], reproducing the same range of production and morphology as large-scale bioreactors, making screening easier and facilitating further upscaling.

#### 2.2.3. Macroscopic Morphology Conditions, Programmed Cell Death and Second Mycelium Differentiation in Liquid Cultures

PCD is the key event that triggers the differentiation of the antibiotic producer, mycelium (MII), in liquid and solid cultures [2]. However, the specific signals derived from cell death are not yet known. The production of *N*-acetylglucosamine from peptidoglycan dismantling accelerates development and antibiotic production [67,68] and might be one of the signals released during PCD.

A simple methodology based on fluorometric measures of cultures stained with SYTO9 and propidium iodide was designed to quantify PCD in liquid cultures [69]. This method allows the efficiency of antibiotic production to be predicted based on the level of PCD [69].

Strains showing dispersed growth take a long time to suffer PCD, and sometimes, PCD does not occur. Modify the developmental conditions to enhance PCD and MII differentiation, leads to an improvement in secondary metabolite production. This approach was recently applied to enhance flavonoid production in a strain of *Streptomyces albus* [15] and to enhance microbial transglutaminase production from *Streptomyces mobaraensis* [14]. The “PCD-MII” approach complements other approaches well; there is no secondary metabolite production without differentiation of MII, but there are biosynthetic pathways that in addition to MII differentiation, need specific elicitors to become active [70].

### 2.3. L-Forms

An interesting alternative that would avoid the problems of mycelial growth in industry, is the use of L-forms, which are individual cells without cell walls [71]. However, until now, the antibiotic levels reached by *Streptomyces* L-forms have been quite minor compared to those reached by the regular form. Therefore, future research should explore whether L-forms could offer an industrial alternative.

### 2.4. Other Strategies

A big challenge in screening for new secondary metabolites is exploring non-cultivated bacteria. The scientific community is aware of the huge quantity of microorganisms that are not cultivated under laboratory conditions. Next Generation Sequencing revealed the big pharmacological potential of uncultured bacteria. Innovative culturing techniques, such as the isolation chip (iChip), are being used successfully in combination with co-cultures to grow previously uncultured bacteria [72]. The study of unexplored niches to look for new Actinomycetes is another strategy that enables the discovery of new species and compounds [73,74,75]. The combination of these two methods is a promising strategy to identify new compounds.

One of the newest strategies focusses on primary metabolism and vegetative growth. Very recently, work by Schniete et al. [76] showed how genetic redundancy within actinobacterial genomes allows functional specialization of two pyruvate kinases in *Streptomyces* under different life cycle stages and environmental conditions. Genetic redundancy within actinobacteria genomes as being a key to understanding how the plasticity of this microorganism enhances the production of clinically useful molecules. Furthermore, Cihak et al. [77] recently described the production of secondary metabolites during germination in *Streptomyces coelicolor*. The germination stage was ignored in most secondary metabolite screening campaigns and constitutes a potential source of bioactive compounds to be explored [77].

## 3. Conclusions

We generally face the great challenge of fighting antibiotic resistance, which is growing much faster than our capacity to find new antimicrobials and new strategies to face this problem. The *Streptomyces* genus is still a huge source of natural bioactive compounds, but we need to form new strategies to avoid rediscovering compounds. There is not a single methodology to trigger differentiation, activate cryptic secondary metabolism pathways and improve the discovery of new bioactive compounds. However, the multidisciplinary biosynthetic cluster specific and non-specific approaches discussed in this manuscript, will be key to improving the screening for new secondary metabolites from *streptomycetes*.

## Figures and Tables

**Table 1:** Non-specific methods and some successful examples of their enforcement. “Enhance” means an improvement in production; “cryptic” means activation of the expression of cryptic pathways.

Methods	Microorganism	Product	Effect	Ref.
**Media manipulation**	*S. roseosporus*	Daptomycin	Enhance	[78]
**Stress Response**	*S. venezuelae*	Jadomycin B	Enhance	[79]
	*S. hygroscopicus*	Validamycin A	Enhance	[21]
	*S. parvulus*	Manumycin family	Cryptic	[80]
	*S. coelicolor*	Ectoine, 5-hydroxyectoine	Enhance	[81]
	*S. coelicolor*	Methylenomycin	Enhance	[82]
**One Strain Many Compounds (OSMAC)**	*S. parvulus*	20 cryptic compounds	Cryptic	[80]
**Random Mutagenesis**	*S. clavuligerus*	Clavulanic acid	Enhance	[83]
	*S. hygroscopicus*	Rapamycin	Enhance	[84]
	*S. coelicolor*	Actinorhodin, Undecylprodigiosin	Enhance	[22]
**Ribosomal Engineering**	*S. coelicolor*	Actinorhodin	Enhance	[85]
**Engineering Global Regulation**	*S. coelicolor*	Actinorhodin, Prodigiosin, Calcium-Dependent Antibiotic	Enhance	[86]
	*S. griseus*	Streptomycin	Enhance	[86]
	*S. griseochromogenes*	Blasticidin S	Enhance	[86]
**Elicitors**	*S. coelicolor*	Actinorhodin	Enhance	[87]
	*S. pristinaespiralis*	Desferrioxamine B/E	Enhance	[20]
	*S. peucetius*	Doxorubicin, Baumycin	Enhance	[20]
	*S. coelicolor*	Actinorhodin, Undecylprodigiosin	Enhance	[68]
	*S. lividans*	Prodiginine	Enhance	[88]
	*S. griseus*	Streptomycin	Enhance	[21]
	*S. natalensis*	Pimaricin	Enhance	[89]
	29 strains	Cryptic compounds	Cryptic	[45]
**Metabolic Engineering**	*S. clavuligerus*	FK606	Enhance	[90]
	*S. coelicolor*	Actinorhodin	Enhance	[90]
	*S. rimosus*	Oxytetracycline	Enhance	[91]
**Co-cultures**	*S. rimosus* MY02	Antifungal activity	Enhance	[36]
	*S. coelicolor*	Actinorhodin	Enhance	[37]
	*S. fradiae 007*	Phenolic polyketides	Enhance	[38]
	Marine *streptomycetes*	See tables in reference	Cryptic	[39]
**Conditioning Morphology (PCD + MII)**	*S. cattleya*	Tienamycin	Enhance	[92]
	*S. cinereoruber*	Rodomycin	Enhance	
	*Saccharopolyspora erythraea*	Erithromycin	Enhance	
	*S. coelicolor*	Actinorhodin	Enhance	

**Table 2:** Biosynthetic cluster specific methods and some successful examples of their enforcement.

Methods	Microorganism	Product	Effect	Ref.
**Engineering Self-Resistance**	*S. peucetius*	Doxorubicin, Daunorubicin	Enhance	[93]
	*S. avermitilis*	Avermectin,	Enhance	[94]
	*S. coelicolor*	Actinorhodin	Enhance	[95]
**Regulatory Engineering**				
Delete repressor AbsA2~P	*S. coelicolor*	Actinorhodin, Undecylprodigiosin, Calcium-dependent antibiotic	Enhance	[96]
Overexpress AverR/StrR	*S. avermitilis*	Avermectin	Enhance	[97]
Overexpress AverR/StrR	*S. griseous*	Streptomycin	Enhance	[98]
Overexpress SamR0484	*S. ambofaciens*	Stambomicin A-D	Cryptic	[99]
Delete repressor *cmmRII*	*S. griseus*	Chromomycin	Enhance	[100]
Delete repressor AlpW	*S. ambofaciens*	Alpomycin	Enhance	[101]
**Heterologous Expression**	*S. avermitilis*	Streptomycin	Enhance	[102]
	*S. coelicolor*	Chloramphenicol	Enhance	[103]
	*S. coelicolor*	Congocidine	Enhance	[103]
	*S. cyaneus*	CECT 3335 laccase	Enhance	[27]
	*S. lividans TK24*	Mithramycin A	Enhance	[104]
	*Streptomyces* sp.	Neothioviridamide	Cryptic	[105]
	Several wild-type	Siamycin-I	Cryptic	[29]
**Combinatorial Biosynthesis**	*S. albus J1074*	Novel paulomycin	Cryptic	[31]
	See table 1 in ref.			[30]
**Conditioning Morphology (PCD + MII)**	*S. albus*	Apigenin, Luteolin	Enhance	[15]
	*S. mobarensis*	Microbial transglutaminase	Enhance	[14]

## References

[1] Worrall J.A., Vijgenboom E. (2010). Copper mining in *streptomyces*: Enzymes, natural products and development. Nat. Prod. Rep..

[2] Manteca A., Alvarez R., Salazar N., Yague P., Sanchez J. (2008). Mycelium differentiation and antibiotic production in submerged cultures of *Streptomyces coelicolor*. Appl. Environ. Microbiol..

[3] Yague P., Willemse J., Koning R.I., Rioseras B., Lopez-Garcia M.T., Gonzalez-Quinonez N., Lopez-Iglesias C., Shliaha P.V., Rogowska-Wrzesinska A., Koster A.J. (2016). Subcompartmentalization by cross-membranes during early growth of *streptomyces* hyphae. Nat. Commun..

[4] Manteca A., Fernandez M., Sanchez J. (2005). A death round affecting a young compartmentalized mycelium precedes aerial mycelium dismantling in confluent surface cultures of *streptomyces* antibioticus. Microbiology.

[5] Flardh K. (2003). Growth polarity and cell division in *streptomyces*. Curr. Opin. Microbiol..

[6] Manteca A., Jung H.R., Schwammle V., Jensen O.N., Sanchez J. (2010). Quantitative proteome analysis of *Streptomyces coelicolor* nonsporulating liquid cultures demonstrates a complex differentiation process comparable to that occurring in sporulating solid cultures. J. Proteome Res..

[7] Yague P., Rodriguez-Garcia A., Lopez-Garcia M.T., Rioseras B., Martin J.F., Sanchez J., Manteca A. (2014). Transcriptomic analysis of liquid non-sporulating *Streptomyces coelicolor* cultures demonstrates the existence of a complex differentiation comparable to that occurring in solid sporulating cultures. PLoS ONE.

[8] Giudici R., Pamboukian C.R., Facciotti M.C. (2004). Morphologically structured model for antitumoral retamycin production during batch and fed-batch cultivations of streptomyces olindensis. Biotechnol. Bioeng..

[9] Vecht-Lifshitz S.E., Sasson Y., Braun S. (1992). Nikkomycin production in pellets of streptomyces tendae. J. Appl. Bacteriol..

[10] Sarra M., Casas C., Godia F. (1997). Continuous production of a hybrid antibiotic by *Streptomyces lividans* TK21 pellets in a three-phase fluidized-bed bioreactor. Biotechnol. Bioeng..

[11] Jonsbu E., McIntyre M., Nielsen J. (2002). The influence of carbon sources and morphology on nystatin production by streptomyces noursei. J. Biotechnol..

[12] Park Y., Tamura S., Koike Y., Toriyama M., Okabe M. (1997). Mycelial pellet intrastructure visualization and viability prediction in a culture of streptomyces fradiae using confocal scanning laser microscopy. J. Ferment. Bioeng..

[13] Rioseras B., Lopez-Garcia M.T., Yague P., Sanchez J., Manteca A. (2014). Mycelium differentiation and development of *Streptomyces coelicolor* in lab-scale bioreactors: Programmed cell death, differentiation, and lysis are closely linked to undecylprodigiosin and actinorhodin production. Bioresour. Technol..

[14] Treppiccione L., Ottombrino A., Luongo D., Maurano F., Manteca A., Lombó F., Rossi M. (2017). Development of gluten with immunomodulatory properties using mTG-active food grade supernatants from *Streptomyces mobaraensis* cultures. J. Funct. Foods.

[15] Marin L., Gutierrez-Del-Rio I., Yague P., Manteca A., Villar C.J., Lombo F. (2017). De novo biosynthesis of apigenin, luteolin, and eriodictyol in the actinomycete *Streptomyces albus* and production improvement by feeding and spore conditioning. Front. Microbiol..

[16] Zhang L., Zhang L., Han X., Du M., Zhang Y., Feng Z., Yi H., Zhang Y. (2012). Enhancement of transglutaminase production in *Streptomyces mobaraensis* as achieved by treatment with excessive MgCl_2_. Appl. Microbiol. Biotechnol..

[17] Onaka H. (2017). Novel antibiotic screening methods to awaken silent or cryptic secondary metabolic pathways in actinomycetes. J. Antibiot..

[18] Genilloud O. (2014). The re-emerging role of microbial natural products in antibiotic discovery. Antonie Van Leeuwenhoek.

[19] Genilloud O. (2017). Actinomycetes: Still a source of novel antibiotics. Nat. Prod. Rep..

[20] Craney A., Ahmed S., Nodwell J. (2013). Towards a new science of secondary metabolism. J. Antibiot..

[21] Yoon V., Nodwell J.R. (2014). Activating secondary metabolism with stress and chemicals. J. Ind. Microbiol. Biotechnol..

[22] Xu Z., Wang Y., Chater K.F., Ou H.Y., Xu H.H., Deng Z., Tao M. (2017). Large-scale transposition mutagenesis of *Streptomyces coelicolor* identifies hundreds of genes influencing antibiotic biosynthesis. Appl. Environ. Microbiol..

[23] Khaliq S., Akhtar K., Afzal Ghauri M., Iqbal R., Mukhtar Khalid A., Muddassar M. (2009). Change in colony morphology and kinetics of tylosin production after UV and gamma irradiation mutagenesis of streptomyces fradiae NRRL-2702. Microbiol. Res..

[24] Korbekandi H., Darkhal P., Hojati Z., Abedi D., Hamedi J., Pourhosein M. (2010). Overproduction of clavulanic acid by UV mutagenesis of *Streptomyces clavuligerus*. Iran. J. Pharm. Res..

[25] Hosaka T., Ohnishi-Kameyama M., Muramatsu H., Murakami K., Tsurumi Y., Kodani S., Yoshida M., Fujie A., Ochi K. (2009). Antibacterial discovery in actinomycetes strains with mutations in RNA polymerase or ribosomal protein S12. Nat. Biotechnol..

[26] Ahmed S., Craney A., Pimentel-Elardo S.M., Nodwell J.R. (2013). A synthetic, speciesspecific activator of secondary metabolism and sporulation in *Streptomyces coelicolor*. Chembiochem.

[27] Ece S., Lambertz C., Fischer R., Commandeur U. (2017). Heterologous expression of a *Streptomyces cyaneus* laccase for biomass modification applications. AMB Express.

[28] Baltz R.H. (2010). Streptomyces and saccharopolyspora hosts for heterologous expression of secondary metabolite gene clusters. J. Ind. Microbiol. Biotechnol..

[29] Daniel-Ivad M., Hameed N., Tan S., Dhanjal R., Socko D., Pak P., Gverzdys T., Elliot M.A., Nodwell J.R. (2017). An engineered allele of afsQ1 facilitates the discovery and investigation of cryptic natural products. ACS Chem. Biol..

[30] Olano C., Mendez C., Salas J.A. (2010). Post-PKS tailoring steps in natural product-producing actinomycetes from the perspective of combinatorial biosynthesis. Nat. Prod. Rep..

[31] González A., Rodríguez M., Braña A.F., Méndez C., Salas J.A., Olano C. (2016). New insights into paulomycin biosynthesis pathway in *Streptomyces albus* j1074 and generation of novel derivatives by combinatorial biosynthesis. Microb. Cell Fact..

[32] Baltz R.H. (2017). Synthetic biology, genome mining, and combinatorial biosynthesis of NRPS-derived antibiotics: A perspective. J. Ind. Microbiol. Biotechnol..

[33] Wang W., Ji J., Li X., Wang J., Li S., Pan G., Fan K., Yang K. (2014). Angucyclines as signals modulate the behaviors of *Streptomyces coelicolor*. Proc. Natl. Acad. Sci. USA.

[34] Marmann A., Aly A.H., Lin W., Wang B., Proksch P. (2014). Co-cultivation—A powerful emerging tool for enhancing the chemical diversity of microorganisms. Mar. Drugs.

[35] Piel J. (2004). Metabolites from symbiotic bacteria. Nat. Prod. Rep..

[36] Yu J., Liu Q., Chen C., Qi X. (2017). Antifungal activity change of streptomyces rimosus MY02 mediated by confront culture with other microorganism. J. Basic Microbiol..

[37] Perez J., Munoz-Dorado J., Brana A.F., Shimkets L.J., Sevillano L., Santamaria R.I. (2011). *Myxococcus Xanthus* induces actinorhodin overproduction and aerial mycelium formation by *Streptomyces coelicolor*. Microb. Biotechnol..

[38] Wang Y., Wang L., Zhuang Y., Kong F., Zhang C., Zhu W. (2014). Phenolic polyketides from the co-cultivation of marine-derived *Penicillium* sp. Wc-29-5 and *Streptomyces fradiae* 007. Mar. Drugs.

[39] Sung A.A., Gromek S.M., Balunas M.J. (2017). Upregulation and identification of antibiotic activity of a marine-derived *Streptomyces* sp. Via co-cultures with human pathogens. Mar. Drugs.

[40] Wang D., Yuan J., Gu S., Shi Q. (2013). Influence of fungal elicitors on biosynthesis of natamycin by streptomyces natalensis HW-2. Appl. Microbiol. Biotechnol..

[41] Sharma R., Jamwal V., Singh V.P., Wazir P., Awasthi P., Singh D., Vishwakarma R.A., Gandhi S.G., Chaubey A. (2017). Revelation and cloning of valinomycin synthetase genes in streptomyces lavendulae acr-da1 and their expression analysis under different fermentation and elicitation conditions. J. Biotechnol..

[42] Gubbens J., Janus M.M., Florea B.I., Overkleeft H.S., van Wezel G.P. (2017). Identification of glucose kinase-dependent and -independent pathways for carbon control of primary metabolism, development and antibiotic production in *Streptomyces coelicolor* by quantitative proteomics. Mol. Microbiol..

[43] Park S.S., Yang Y.H., Song E., Kim E.J., Kim W.S., Sohng J.K., Lee H.C., Liou K.K., Kim B.G. (2009). Mass spectrometric screening of transcriptional regulators involved in antibiotic biosynthesis in *Streptomyces coelicolor* A3(2). J. Ind. Microbiol. Biotechnol..

[44] Liu G., Chater K.F., Chandra G., Niu G., Tan H. (2013). Molecular regulation of antibiotic biosynthesis in streptomyces. Microbiol. Mol. Biol. Rev..

[45] Pimentel-Elardo S.M., Sorensen D., Ho L., Ziko M., Bueler S.A., Lu S., Tao J., Moser A., Lee R., Agard D. (2015). Activity-independent discovery of secondary metabolites using chemical elicitation and cheminformatic inference. ACS Chem. Biol..

[46] Van Dissel D., Claessen D., Roth M., van Wezel G.P. (2015). A novel locus for mycelial aggregation forms a gateway to improved streptomyces cell factories. Microb. Cell Fact..

[47] Chaplin A.K., Petrus M.L., Mangiameli G., Hough M.A., Svistunenko D.A., Nicholls P., Claessen D., Vijgenboom E., Worrall J.A. (2015). Glxa is a new structural member of the radical copper oxidase family and is required for glycan deposition at hyphal tips and morphogenesis of *Streptomyces lividans*. Biochem. J..

[48] Petrus M.L., Vijgenboom E., Chaplin A.K., Worrall J.A., van Wezel G.P., Claessen D. (2016). The dyp-type peroxidase dtpa is a tat-substrate required for glxa maturation and morphogenesis in streptomyces. Open Biol..

[49] Petrus M.L., Claessen D. (2014). Pivotal roles for streptomyces cell surface polymers in morphological differentiation, attachment and mycelial architecture. Antonie Van Leeuwenhoek.

[50] Susstrunk U., Pidoux J., Taubert S., Ullmann A., Thompson C.J. (1998). Pleiotropic effects of camp on germination, antibiotic biosynthesis and morphological development in *Streptomyces coelicolor*. Mol. Microbiol..

[51] De Jong W., Manteca A., Sanchez J., Bucca G., Smith C.P., Dijkhuizen L., Claessen D., Wosten H.A. (2009). Nepa is a structural cell wall protein involved in maintenance of spore dormancy in *Streptomyces coelicolor*. Mol. Microbiol..

[52] Bobek J., Strakova E., Zikova A., Vohradsky J. (2014). Changes in activity of metabolic and regulatory pathways during germination of *S. coelicolor*. BMC Genomics.

[53] Rioseras B., Yague P., Lopez-Garcia M.T., Gonzalez-Quinonez N., Binda E., Marinelli F., Manteca A. (2016). Characterization of SCO4439, a d-alanyl-d-alanine carboxypeptidase involved in spore cell wall maturation, resistance, and germination in *Streptomyces coelicolor*. Sci. Rep..

[54] Sexton D.L., St-Onge R.J., Haiser H.J., Yousef M.R., Brady L., Gao C., Leonard J., Elliot M.A. (2015). Resuscitation-promoting factors are Cell wall-lytic enzymes with important roles in the germination and growth of *Streptomyces coelicolor*. J. Bacteriol..

[55] Noens E.E., Mersinias V., Willemse J., Traag B.A., Laing E., Chater K.F., Smith C.P., Koerten H.K., van Wezel G.P. (2007). Loss of the controlled localization of growth stage-specific cell-wall synthesis pleiotropically affects developmental gene expression in an ssga mutant of *Streptomyces coelicolor*. Mol. Microbiol..

[56] Zacchetti B., Willemse J., Recter B., van Dissel D., van Wezel G.P., Wosten H.A., Claessen D. (2016). Aggregation of germlings is a major contributing factor towards mycelial heterogeneity of *streptomyces*. Sci. Rep..

[57] Girard G., Traag B.A., Sangal V., Mascini N., Hoskisson P.A., Goodfellow M., van Wezel G.P. (2013). A novel taxonomic marker that discriminates between morphologically complex actinomycetes. Open Biol..

[58] Ronnest N.P., Stocks S.M., Lantz A.E., Gernaey K.V. (2012). Comparison of laser diffraction and image analysis for measurement of *Streptomyces coelicolor* cell clumps and pellets. Biotechnol. Lett..

[59] Van Veluw G.J., Petrus M.L., Gubbens J., de Graaf R., de Jong I.P., van Wezel G.P., Wosten H.A., Claessen D. (2012). Analysis of two distinct mycelial populations in liquid-grown streptomyces cultures using a flow cytometry-based proteomics approach. Appl. Microbiol. Biotechnol..

[60] Petrus M.L., van Veluw G.J., Wosten H.A., Claessen D. (2014). Sorting of streptomyces cell pellets using a complex object parametric analyzer and sorter. J. Vis. Exp..

[61] Willemse J., Buke F., van Dissel D., Grevink S., Claessen D., van Wezel G.P. (2017). Sparticle, an algorithm for the analysis of filamentous microorganisms in submerged cultures. Antonie Van Leeuwenhoek.

[62] Celler K., Cristian P., van Loosdrecht M.C., van Wezel G.P. (2012). Structured morphological modeling as a framework for rational strain design of streptomyces species. Antonie Van Leeuwenhoek.

[63] Nieminen L., Webb S., Smith M.C., Hoskisson P.A. (2013). A flexible mathematical model platform for studying branching networks: Experimentally validated using the model actinomycete, *Streptomyces coelicolor*. PLoS ONE.

[64] Van Dissel D., Claessen D., van Wezel G.P. (2014). Morphogenesis of streptomyces in submerged cultures. Adv. Appl. Microbiol..

[65] Ha S., Lee K.J., Lee S.I., Gwak H.J., Lee J.H., Kim T.W., Choi H.J., Jang J.Y., Choi J.S., Kim C.J. (2017). Optimization of herbicidin a production in submerged culture of streptomyces scopuliridis m40. J. Microbiol. Biotechnol..

[66] Van Dissel D., van Wezel G.P. (2017). Morphology-driven downscaling of streptomyces lividans to micro-cultivation. Antonie Van Leeuwenhoek.

[67] Rigali S., Nothaft H., Noens E.E., Schlicht M., Colson S., Müller M., Joris B., Koerten H.K., Hopwood D.A., Titgemeyer F., van Wezel G.P. (2006). The sugar phosphotransferase system of *Streptomyces coelicolor* is regulated by the gntr-family regulator dasr and links n-acetylglucosamine metabolism to the control of development. Mol. Microbiol..

[68] Rigali S., Titgemeyer F., Barends S., Mulder S., Thomae A.W., Hopwood D.A., van Wezel G.P. (2008). Feast or famine: The global regulator dasr links nutrient stress to antibiotic production by streptomyces. EMBO Rep..

[69] Yague P., Manteca A., Simon A., Diaz-Garcia M.E., Sanchez J. (2010). New method for monitoring programmed cell death and differentiation in submerged streptomyces cultures. Appl. Environ. Microbiol..

[70] Yague P., Lopez-Garcia M.T., Rioseras B., Sanchez J., Manteca A. (2013). Pre-sporulation stages of streptomyces differentiation: State-of-the-art and future perspectives. FEMS Microbiol. Lett..

[71] Innes C.M., Allan E.J. (2001). Induction, growth and antibiotic production of streptomyces viridifaciens l-form bacteria. J. Appl. Microbiol..

[72] Kealey C., Creaven C.A., Murphy C.D., Brady C.B. (2017). New approaches to antibiotic discovery. Biotechnol. Lett..

[73] Bai L., Liu C., Guo L., Piao C., Li Z., Li J., Jia F., Wang X., Xiang W. (2016). *Streptomyces formicae* sp. Nov., a novel actinomycete isolated from the head of *Camponotus japonicus* mayr. Antonie Van Leeuwenhoek.

[74] Sarmiento-Vizcaino A., Brana A.F., Gonzalez V., Nava H., Molina A., Llera E., Fiedler H.P., Rico J.M., Garcia-Florez L., Acuna J.L. (2016). Atmospheric dispersal of bioactive *Streptomyces albidoflavus* strains among terrestrial and marine environments. Microb. Ecol..

[75] Sarmiento-Vizcaino A., Gonzalez V., Brana A.F., Palacios J.J., Otero L., Fernandez J., Molina A., Kulik A., Vazquez F., Acuna J.L. (2017). Pharmacological potential of phylogenetically diverse actinobacteria isolated from deep-sea coral ecosystems of the submarine aviles canyon in the cantabrian sea. Microb. Ecol..

[76] Schniete J.K., Cruz-Morales P., Selem-Mojica N., Fernandez-Martinez L.T., Hunter I.S., Barona-Gomez F., Hoskisson P.A. (2018). Expanding primary metabolism helps generate the metabolic robustness to facilitate antibiotic biosynthesis in streptomyces. MBio.

[77] Cihak M., Kamenik Z., Smidova K., Bergman N., Benada O., Kofronova O., Petrickova K., Bobek J. (2017). Secondary metabolites produced during the germination of *Streptomyces coelicolor*. Front. Microbiol..

[78] Yu G., Jia X., Wen J., Lu W., Wang G., Caiyin Q., Chen Y. (2011). Strain improvement of streptomyces roseosporus for daptomycin production by rational screening of he-ne laser and NTG induced mutants and kinetic modeling. Appl. Biochem. Biotechnol..

[79] Jakeman D.L., Graham C.L., Young W., Vining L.C. (2006). Culture conditions improving the production of jadomycin b. J. Ind. Microbiol. Biotechnol..

[80] Bode H.B., Bethe B., Hofs R., Zeeck A. (2002). Big effects from small changes: Possible ways to explore nature’s chemical diversity. Chembiochem.

[81] Bursy J., Kuhlmann A.U., Pittelkow M., Hartmann H., Jebbar M., Pierik A.J., Bremer E. (2008). Synthesis and uptake of the compatible solutes ectoine and 5-hydroxyectoine by *Streptomyces coelicolor* A3(2) in response to salt and heat stresses. Appl. Environ. Microbiol..

[82] Hayes A., Hobbs G., Smith C.P., Oliver S.G., Butler P.R. (1997). Environmental signals triggering methylenomycin production by *Streptomyces coelicolor* A3(2). J. Bacteriol..

[83] Medema M.H., Alam M., Breitling R., Takano E. (2011). The future of industrial antibiotic production: From random mutagenesis to synthetic biology. Bioeng. Bugs..

[84] Cheng Y.R., Huang J., Qiang H., Lin W.L., Demain A.L. (2001). Mutagenesis of the rapamycin producer streptomyces hygroscopicus FC904. J. Antibiot..

[85] Wang G., Hosaka T., Ochi K. (2008). Dramatic activation of antibiotic production in *Streptomyces coelicolor* by cumulative drug resistance mutations. Appl. Environ. Microbiol..

[86] McKenzie N.L., Thaker M., Koteva K., Hughes D.W., Wright G.D., Nodwell J.R. (2010). Induction of antimicrobial activities in heterologous streptomycetes using alleles of the *Streptomyces coelicolor* gene absa1. J. Antibiot..

[87] Foley T.L., Young B.S., Burkart M.D. (2009). Phosphopantetheinyl transferase inhibition and secondary metabolism. FEBS J..

[88] Onaka H. (2009). Biosynthesis of indolocarbazole and goadsporin, two different heterocyclic antibiotics produced by actinomycetes. Biosci. Biotechnol. Biochem..

[89] Recio E., Colinas A., Rumbero A., Aparicio J.F., Martin J.F. (2004). Pi factor, a novel type quorum-sensing inducer elicits pimaricin production in streptomyces natalensis. J. Biol. Chem..

[90] Mo S., Ban Y.H., Park J.W., Yoo Y.J., Yoon Y.J. (2009). Enhanced FK506 production in *Streptomyces clavuligerus* ckd1119 by engineering the supply of methylmalonyl-coa precursor. J. Ind. Microbiol. Biotechnol..

[91] Liu Z., Guo M., Qian J., Zhuang Y., Zhang S. (2008). Disruption of ZWF2 gene to improve oxytetraclyline biosynthesis in streptomyces rimosus M4018. Wei Sheng Wu Xue Bao.

[92] Yagüe P., Manteca A. (2018). Optimization of the antibiotic producction in different Streptomyces strains by conditioning the MII differentiation.

[93] Malla S., Niraula N.P., Liou K., Sohng J.K. (2010). Self-resistance mechanism in streptomyces peucetius: Overexpression of drra, drrb and drrc for doxorubicin enhancement. Microbiol. Res..

[94] Qiu J., Zhuo Y., Zhu D., Zhou X., Zhang L., Bai L., Deng Z. (2011). Overexpression of the abc transporter avtab increases avermectin production in streptomyces avermitilis. Appl. Microbiol. Biotechnol..

[95] Xu Y., Willems A., Au-Yeung C., Tahlan K., Nodwell J.R. (2012). A two-step mechanism for the activation of actinorhodin export and resistance in *Streptomyces coelicolor*. MBio.

[96] McKenzie N.L., Nodwell J.R. (2007). Phosphorylated absa2 negatively regulates antibiotic production in *Streptomyces coelicolor* through interactions with pathway-specific regulatory gene promoters. J. Bacteriol..

[97] Guo J., Zhao J., Li L., Chen Z., Wen Y. (2010). Li, J. The pathway-specific regulator aver from *Streptomyces avermitilis* positively regulates avermectin production while it negatively affects oligomycin biosynthesis. Mol. Genet. Genomics.

[98] Retzlaff L., Distler J. (1995). The regulator of streptomycin gene expression, strr, of streptomyces griseus is a DNA binding activator protein with multiple recognition sites. Mol. Microbiol..

[99] Laureti L., Song L., Huang S., Corre C., Leblond P., Challis G.L., Aigle B. (2011). Identification of a bioactive 51-membered macrolide complex by activation of a silent polyketide synthase in *Streptomyces ambofaciens*. Proc. Natl. Acad. Sci. USA.

[100] Menendez N., Brana A.F., Salas J.A., Mendez C. (2007). Involvement of a chromomycin abc transporter system in secretion of a deacetylated precursor during chromomycin biosynthesis. Microbiology.

[101] Bunet R., Song L., Mendes M.V., Corre C., Hotel L., Rouhier N., Framboisier X., Leblond P., Challis G.L., Aigle B. (2010). Characterization and manipulation of the pathway-specific late regulator alpw reveals *Streptomyces ambofaciens* as a new producer of kinamycins. J. Bacteriol..

[102] Gomez-Escribano J.P., Bibb M.J. (2011). Engineering *Streptomyces coelicolor* for heterologous expression of secondary metabolite gene clusters. Microb. Biotechnol..

[103] Komatsu M., Uchiyama T., Omura S., Cane D.E., Ikeda H. (2010). Genome-minimized streptomyces host for the heterologous expression of secondary metabolism. Proc. Natl. Acad. Sci. USA.

[104] Novakova R., Nunez L.E., Homerova D., Knirschova R., Feckova L., Rezuchova B., Sevcikova B., Menendez N., Moris F., Cortes J. (2018). Increased heterologous production of the antitumoral polyketide mithramycin a by engineered streptomyces lividans TK24 strains. Appl. Microbiol. Biotechnol..

[105] Kawahara T., Izumikawa M., Kozone I., Hashimoto J., Kagaya N., Koiwai H., Komatsu M., Fujie M., Sato N., Ikeda H. (2018). Neothioviridamide, a polythioamide compound produced by heterologous expression of a *Streptomyces* sp. Cryptic ripp biosynthetic gene cluster. J. Nat. Prod..

